# Avian Immunoglobulin Y Antibodies Targeting the Protruding or Shell Domain of Norovirus Capsid Protein Neutralize Norovirus Replication in the Human Intestinal Enteroid System

**DOI:** 10.3390/vaccines13121228

**Published:** 2025-12-05

**Authors:** Ming Xia, Mohamed Ichou, Mathew Landivar, Peng Zhou, Sai Navya Vadlamudi, Alice Leruth, Charlotte Nyblade, Paul Cox, Lijuan Yuan, Julius Goepp, Ming Tan

**Affiliations:** 1 Division of Infectious Diseases, Cincinnati Children’s Hospital Medical Center, Cincinnati, OH 45229, USA; xiamingus@yahoo.com; 2 Evimero, 100 Rice Mine Road Loop, Suite 301, Tuscaloosa, AL 35406, USA; mohaitichou@aim.com (M.I.); pcox@evimero-research.com (P.C.); 3 Department of Biomedical Sciences and Pathobiology, Virginia-Maryland College of Veterinary Medicine, Virginia Tech, Blacksburg, VA 24060, USA; mathewdl@vt.edu (M.L.); peng.zhou4975@jacks.sdstate.edu (P.Z.); sainavyav22@vt.edu (S.N.V.); acleruth@vt.edu (A.L.); charlottejn@vt.edu (C.N.); lyuan@vt.edu (L.Y.); 4 Department of Pediatrics, University of Cincinnati College of Medicine, Cincinnati, OH 45229, USA

**Keywords:** norovirus, norovirus protruding (P) domain, norovirus shell (S) domain, avian immunoglobulin Y (IgY), human intestinal enteroid model, passive immunotherapy

## Abstract

Background: Norovirus is a leading cause of epidemic acute gastroenteritis worldwide, associated with significant morbidity, mortality, and economic loss. Despite its global impact, no licensed vaccine is currently available, and vaccine development remains challenging. Methods: We explored avian immunoglobulin Y (IgY) antibodies as a low-cost countermeasure against norovirus infection. We generated recombinant protruding (P) domain proteins from the capsid protein (VP1) of noroviruses, representing two GII.4 variants and the GII.6 genotype. These were combined into a single immunogen to immunize laying hens to produce norovirus VP1-specific IgY antibodies. Results: Immunization of laying hens with the P domain proteins elicited high-titer (>1:450,000) P domain-specific IgY antibodies. The yolk-derived IgY effectively inhibited binding of various norovirus P particles to their histo-blood group antigen ligands, with 50% blocking titers (BT50) up to 1:8533 against homotypic GII.4 and 1:667 against heterotypic G1.1 Norwalk virus P particles. Importantly, the IgY neutralized replication of GII.4 norovirus in the human intestinal enteroid (HIE) system at a high titer of over 1:2500, equivalent to 0.70 µg/mL of total IgY. We also produced norovirus shell (S) domain proteins and corresponding IgY antibodies, which neutralized GII.4 norovirus replication in the HIE model at a titer of ~1:800, equivalent to 2.98 µg/mL of total IgY. This provides the first evidence that the S domain contains neutralizing epitopes. Conclusions: Our findings support the potential of IgY targeting norovirus P or S domains as a scalable, cost-effective strategy for preventing norovirus infection and disease.

## 1. Introduction

Noroviruses are single-stranded, positive-sense RNA viruses belonging to the *Norovirus* genus within the *Caliciviridae* family [1,2]. They are a leading viral cause of acute gastroenteritis (AGE) worldwide, responsible for hundreds of millions of diarrhea cases each year [3,4]. Noroviruses are highly contagious, transmitted primarily via the fecal-oral route through contaminated food or water, as well as person-to-person contact [5]. This often leads to large AGE outbreaks in closed or semi-closed environments such as cruise ships, childcare centers, long-term care facilities, schools, and restaurants [6]. Although norovirus-associated AGE is typically mild and self-limiting, severe illnesses can occur, particularly among vulnerable populations, including young children [7], the elderly [8,9], and immunocompromised individuals [10]. In some cases, the disease may result in life-threatening complications.

Globally, norovirus-associated AGE is estimated to affect approximately 685 million people each year, representing roughly 18% of total diarrheal disease cases [11,12]. These norovirus infections result in an estimated 212,000 deaths each year, with roughly 99% occurring in low- to middle-income countries (LMICs) [11]. In high-income countries (HICs), norovirus remains a major cause of diarrheal illness, including foodborne outbreaks and community-acquired infections, although mortality rates are significantly lower compared to LMICs. Worldwide, norovirus associated diseases impose an estimated annual economic burden of $4.2 billion in direct healthcare costs and $60.3 billion in wider societal losses [13]. In the United States, the CDC reports that norovirus infections lead to 19 to 21 million cases of illnesses annually, resulting in 2.3 million outpatient visits, 465,000 emergency room visits, 109,000 hospitalizations, and roughly 900 deaths [14].

As single-stranded RNA viruses, noroviruses evolve rapidly, resulting in extensive genetic and antigenic diversity. Multiple strains co-circulate during each epidemic season. According to the Norovirus Sentinel Testing and Tracking (NoroSTAT) network system in the United States, a dramatic increase in norovirus-associated AGE outbreaks has been observed during the 2024–2025 norovirus epidemic season [15]. Specifically, 2630 AGE outbreaks were reported between 1 August 2024, and 4 June 2025, nearly double the 1410 outbreaks reported during the same period the previous year [15].

Further studies demonstrated that this surge in AGE outbreaks is mainly due to the emergence of the GII.17 norovirus genotype, a previously rare strain, as the dominant circulating strain. This genotype has overtaken the historically predominant GII.4 genotype, likely due to the lack of herd immunity to the new GII.17 strain in the general population. For over a decade, from 2011 to 2024, GII.4 strains were predominant, being responsible for over 50% of annual outbreaks. However, in the 2024–2025 season, GII.17 accounted for 75.4% of outbreaks, while GII.4 dropped to just 10.7% [16,17]. These findings underscore the diversity and dynamic nature of norovirus prevalence, posing significant challenges for vaccine development, especially as most current candidates target GII.4 strains.

Despite the global burden of norovirus-associated disease, no commercial vaccine is currently available. Recent clinical trials of the two most advanced vaccine candidates have shown either low to modest efficacy in adults [18,19], or failed to demonstrate protective efficacy in infants [20], one of the most vulnerable populations. These findings raise serious concerns about the feasibility of developing a broadly effective vaccine and highlight the need to explore alternative countermeasures.

Structurally, the non-enveloped norovirus is encompassed by a protein capsid that is composed of a single major capsid protein, referred to as viral protein 1 (VP1). VP1 contains two major domains [21], of which the P domain forms the dimeric protrusions on the viral surface, while the S domain constitutes the icosahedral inner shell of the virus. The dimeric protrusions interact with histo-blood group antigens (HBGAs) to initiate viral infection and are widely recognized as a major neutralizing antigen. However, whether the interior shell contains a neutralizing epitope remains unknown. Both the recombinant P and the S domain protein can self-assemble into subviral particles that can induce strong antibody responses, leading to easy production of P and S domain-specific antibodies at high titers [22,23].

Avian immunoglobulin Y (IgY) is gaining recognition as a promising agent for both the prevention and treatment of intestinal infection. Extracted from the egg yolks of immunized hens, IgY offers several key advantages: it is non-invasive, cost-efficient to produce, and exhibits high specificity toward targeted pathogens and the epitopes of their virulence factors [24]. In this study, we generate IgY antibodies targeting the two major domains of the norovirus capsid protein and evaluate their neutralization effect against norovirus infection and replication utilizing a human intestinal enteroid (HIE) system [25,26]. Our results demonstrated that IgY raised against the P domain proteins achieved high titers and effectively neutralized norovirus replication. Notably, the IgY targeting the S domain also exhibited neutralizing activity, demonstrating the S domain as a neutralizing antigen.

## 2. Materials and Methods

### 2.1. Selected Norovirus P and S Domains

**Figure 1:**
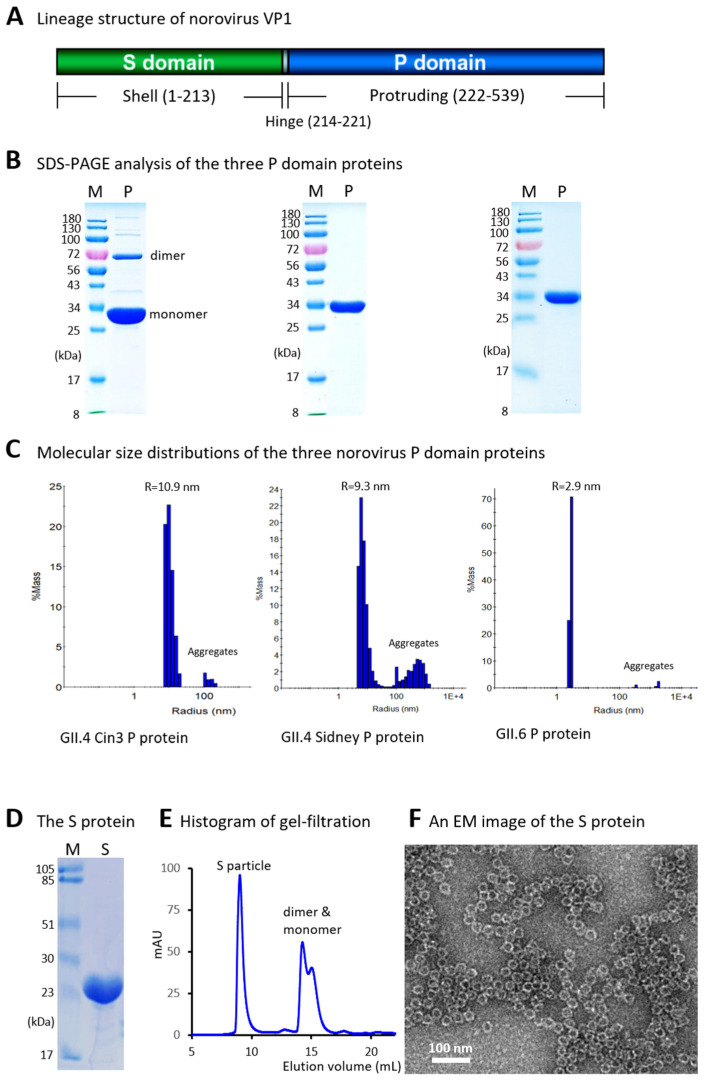
Production and characterization of recombinant protruding (P) and shell (S) domains of the norovirus capsid protein (VP1). (**A**) Schematic representation of the norovirus VP1 protein, showing the S and P domains connected by a short hinge. Amino acid numbering is based on the GII.4 strain VA387. (**B**) SDS-PAGE analysis of the recombinant P domain proteins from GII.4 Cin3 (**left panel**), GII.4 Sydney (**middle panel**), and a GII.6 virus (**right panel**). Lanes M contain protein molecular weight markers (in kDa); lanes P contain the purified P domain proteins. (**C**) Dynamic light scattering (DLS) histograms showing the molecular size distributions of the three P domain proteins. The peak radii (in nanometer, nm) are indicated above each histogram. 1E+4 indicates 1 × 10^4^. (**D**) SDS-PAGE analysis of the purified S domain protein (lane S). (**E**) Gel-filtration chromatography profile showing peaks corresponding to S particles, dimers, and monomers. The Y-axis represents ultraviolet absorbance at 280 nm (A280, mAU), and the X-axis indicates elution volume (in mL). (**F**) Transmission electron micrograph showing typical S particles.

The norovirus P domains, spanning residues E222 to L539 (Figure 1A, based on the VP1 protein of strain VA98387, GenBank accession #: AY038600.3), from three GII noroviruses were selected for this study. These included two GII.4 variants, GII.4 Farmington Hills and GII.4 Sydney, and one GII.6 strain. For the Farmington Hills variant, we selected the Cin 3 virus P domain (GenBank accession #: JQ965810), which is used as the challenge virus in a human challenge model at Cincinnati Children’s Hospital Medical Center. For the Sydney variant, we selected the AB-NOV-GII-4_R82823S3 virus (GenBank accession #: PP661667), and for the GII.6 strain, AB-NOV-GII-6_R82823S2 virus (GenBank accession #: PP661666) was chosen for recombinant P domain protein production. Additionally, the S domain with the hinge region, spanning residues M1 to E221 of the GII.4 strain VA98387 (Figure 1A, GenBank accession #: AY038600.3), was selected for protein production.

### 2.2. Recombinant P and S Domain Protein Production

The three P domain proteins were produced using the Glutathione-S-Transferase (GST) Gene Fusion System (GE Healthcare, Chicago, IL, USA), following previously established protocols [27,28]. Briefly, DNA sequences encoding the P domains, each with a C-terminal cysteine-containing peptide (CDCRGDCFC) to facilitate P particle formation, were synthesized by GenScript and cloned into the plasmid vector pGEX-4T-1 (Creative Biogene, Frankfurt, Germany). The GST-P fusion proteins were expressed in *Escherichia coli* strain BL21 (DE3) and purified using the GST-binding resin (Cytiva Lifesciences, Marlborough, MA, USA). The P domain proteins were subsequently released by thrombin digestion. Due to the low solubility of the GST-P fusion protein of GII.6 norovirus, the C-terminal arginine cluster (RRRAV) of the GII.6 P domain was removed to enhance the solubility. Further purification of the P domain proteins was performed using anion exchange chromatography, as needed, to achieve higher purity. The S domain protein with an R69A mutation, including the hinge region, was produced with a C-terminal Hisx6 tag. The R69A mutation was introduced to remove the trypsin site at this position to protect the recombinant S domain protein from trypsin cleavage.

### 2.3. Sodium Dodecyl Sulfate Polyacrylamide Gel Electrophoresis (SDS-PAGE)

The quality of the purified recombinant proteins was evaluated by SDS-PAGE using 10% to 12% separating gels. To observe the molecular weight of full IgY, the extracted IgY (see below) was treated under non-reducing conditions, without the use of reducing agents (dithiothreitol, DTT and β-mercaptoethanol). Protein concentrations were determined using a NanoDrop spectrophotometer (ThermoFisher Scientific, Waltham, MA, USA). For cross-reference, the protein concentrations were confirmed by SDS-PAGE, using serially diluted bovine serum albumin (BSA, Bio-Rad, Hercules, CA, USA) of known concentrations as standards run on the same gels.

### 2.4. Dynamic Light Scattering (DLS)

DLS was used to evaluate the molecular size distribution of protein samples as described in our previous study [29]. The P domain proteins tend to self-assemble into complexes or particles, which were assessed using the DynaPro^®^ NanoStar^®^ II DLS instrument (WatersTM/Wyatt Technology, Milford, MA, USA). A disposable cuvette and a 10 μL sample volume were used for each measurement. The resulting data were further analyzed using the DYNAMICS software Version 7.1.7.16 (Wyatt Technology, Milford, MA, USA) to generate figures.

### 2.5. Gel-Filtration Chromatography

This procedure was used to assess the self-assembly of S particles from purified S domain proteins using a Fast Performance Liquid Chromatography (FPLC) system (ÄTA pure™ 25 L, GE Healthcare Life Sciences, Chicago, IL, USA). The system was equipped with a size exclusion column (Superdex 200, 10/300 GL, 25 mL bed volume, GE Healthcare Life Sciences, Chicago, IL, USA) for gel-filtration purposes. Elution peaks corresponding to S particles, S dimers, and S monomers were identified by comparison with the elution profiles of previously characterized standards, including the S-HA1 H7 pseudovirus nanoparticle (~3.12 MDa) and GST-dimers (54 kDa). Protein concentrations in the eluate were monitored by measuring absorbance at 280 nm (A280) using a spectrophotometer integrated into the instrument.

### 2.6. Electron Microscopy

The morphology of the S particles self-assembled by the purified S domain protein was examined using negative-stain transmission electron microscopy (TEM). Briefly, purified S domain protein was adsorbed onto grids (FCF200-CV-50, Electron Microscopy Sciences, Hatfield, PA, USA) and stained with 1% ammonium molybdate solution. After air-drying, the grids were examined using a Hitachi electron microscope (model H-7650, Tokyo, Japan) operated at 80 kV, with magnifications ranging from 15,000× to 40,000×.

### 2.7. Immunization of Laying Hens

Commercial White Rock and Rhode Island Red crossbred sex-link hens (Pinola Hatchery, Shippensburg, PA, USA) were immunized as previously reported [30]. Briefly, the three P domain proteins in 20 mM tris buffer (pH 8.0) with 250 mM NaCl were combined in a 1:1:1 ratio (*w*/*w*) to form a single immunogen mixture. This mixture was then emulsified with the poultry adjuvant Montanide ISA 70 VG (Seppic Inc., Fairfield, NJ, USA) at a 30:70 (*v*/*v*) antigen-to-adjuvant ratio, following manufacturer’s instruction. The resulting emulsion, containing 100 μg of the P domain protein per mL, was filtered through a 0.22 μm pore-size polycarbonate filter (VWR International, Radnor, PA, USA). For immunization, 0.5 mL of the antigen/adjuvant preparation, containing 50 μg of antigen, was injected intramuscularly into each breast of two hens (test group) on day 1. Two additional hens served as the unimmunized control group. Two booster immunizations were administered on day 14 and day 28, using the same procedure. The S domain protein was used to immunize two additional hens using the same protocols as for the P domain proteins.

### 2.8. IgY Extraction

Starting one day before the initial immunization, two eggs were collected weekly from each group of hens. IgY was isolated from the yolks using the polyethylene glycol (PEG) method [31]. Briefly, the yolks were pooled, and lipids were removed by centrifugation using 12% PEG 6000 (Alfa Aesar, Haverhill, MA, USA). The precipitate was resuspended in phosphate-buffered saline (PBS, pH 7.4), dialyzed against 0.1% sodium chloride for 16 h, and subsequently against PBS for an additional three hours. Protein concentration was measured using the bicinchoninic acid (BCA) assay kit (Thermo Fisher Scientific, Rockford, IL, USA), with bovine gamma-globulin (Bio-Rad, Hercules, CA, USA) as the standard. The purified IgY was stored at −20 °C.

### 2.9. Norovirus-Specific Antibody Determination

IgY antibody titers against various norovirus P antigens were measured by enzyme-linked immunosorbent assay (ELISA). Briefly, 96-well microtiter plates were coated with the three P domain proteins (100 µL/well at 2 µg/mL), either individually or in combination, as capture antigens. After blocking with 5% nonfat milk in PBS (pH 7.4), serial dilutions of chicken IgY samples were added. Bound antibodies were detected using goat anti-chicken IgY Fc antibody-horseradish peroxidase (HRP) conjugate (Antibodies.com LLC, St. Louis, MO, USA) at a 1:5000 dilution. Following the addition of OptEIA TMB Substrate Reagent Set (BD Biosciences, San Jose, CA, USA), absorbance was measured at 450 nm. IgY isolated from yolks of unimmunized hens served as negative controls. Two non-antigen-coated wells were included on each plate as background (blank) controls. Antigen-specific IgY titers were defined as the highest IgY dilutions yielding an OD450 signal ≥ 0.3. This higher threshold was set due to low background OD detected in the unimmunized IgY.

### 2.10. Blocking Titers Against Norovirus P Particle-HBGA Interaction

Norovirus P domain binds HBGAs on host cell surfaces to initiate infection. An ELISA-based assay has been established as a surrogate neutralization method to evaluate the inhibitory effect of antibodies on this interaction. In this blocking assay, well-characterized saliva samples containing type A HBGAs were coated onto 96-well microtiter plates. Norovirus P particles were pre-incubated with serial dilutions of IgY isolated from egg yolks of hens immunized with norovirus P domain proteins or control samples, and then added to the HBGA-coated wells. A reduction in binding signals, compared to wells without antibody treatment, indicated a blocking effect. The highest IgY dilution resulting in at least a 50% reduction in P particle binding to HBGAs was defined as the 50% blocking titer (BT50).

### 2.11. HIE-Based Norovirus Neutralization Assays

The jejunum derived HIE line J2 used in this study was originally established in the Estes Lab [25]. Cultures were maintained in Matrigel domes and expanded in IntestiCult™ medium (StemCell Technologies, Vancouver, BC, Canada). For differentiation, monolayers were seeded onto collagen IV-coated 96-well plates. Once confluence was achieved, the medium was switched to differentiation medium (StemCell Technologies, Vancouver, BC, Canada) for 5–7 days. Prior to infection with norovirus (GII.4 Sydney variant), viruses containing 5 × 10^5^ viral RNA copies were pre-incubated with sterile IgY antibodies at the indicated dilutions and concentrations at 37 °C for 1 h. The virus-antibody mixtures were then added to differentiated HIE monolayers in the presence of bile acid (500 µM glycochenodeoxycholic acid, GCDCA) and ceramide (50 µM), which enhance norovirus entry into the host cells in the HIE system [32,33]. After a 1 h incubation at 37 °C, the virus mixtures were removed, followed by washing and the addition of fresh medium. The well-characterized norovirus nanobody M4, a single-domain antibody fragment (VHH) derived from camelid heavy-chain antibodies targeting a conserved epitope, away from the HBGA binding site on norovirus capsid P domain, was included as a positive control [34].

At 72 h post-infection (hpi), cells were harvested for viral RNA isolation. Viral replication was quantified by RT-qPCR targeting the ORF1-ORF2 junction of the norovirus genome. The result of each independent experiment represents the average of four to eight identically treated replicate wells. Each data point reflects the mean of at least three independent experiments. Viral replication rates following antibody treatment were calculated by comparing viral RNA copy numbers between antibody-treated and untreated groups, with the untreated group set as 100%. The 50% neutralization titers (NT50) were defined as the highest dilutions of the IgY that resulted in a ≥50% reduction in viral replication compared to the untreated virus-only control. The GII.4 Sydney [P31] virus was kindly provided by Dr. Karina Gomes at the Viral Gastroenteritis Laboratory, Virology Department, Antimicrobial Service of Argentina’s National Institute of Infectious Diseases.

### 2.12. Statistical Analysis

Statistical differences between two data groups were analyzed using an unpaired *t* test in GraphPad Prism 9.0 (GraphPad Software, Inc., San Diego, CA, USA). Differences were considered non-significant (ns) when *p* > 0.05; significant when *p* < 0.05 (marked as “*”); highly significant when *p* < 0.01 (marked as “**”); and extremely significant when *p* < 0.001 (***), or <0.0001 (****), respectively.

## 3. Results

### 3.1. Production and Characterization of Norovirus P Domain Proteins

GST-P domain fusion proteins containing the P domains from the three selected noroviruses, each with a C-terminal cysteine-rich peptide [28], were produced using an *E. coli* expression system (see Section 2). The P domain proteins were released by thrombin digestion, while the GST tag remained bound to the GST-binding resin. SDS-PAGE analysis showed that all three P domain proteins were of high purity and migrated to the expected positions (Figure 1B), corresponding to their calculated molecular weights of approximately 34.5 kDa. Notably, a small proportion of dimeric form (~69 kDa) was observed for the P domain protein of the GII.4 Cin3 virus on the gel (Figure 1B, left panel), consistent with previous findings that the P domain protein of the GII.4 strain VA387 also exhibited dimers in SDS-PAGE [27].

Since native P domain proteins form dimers [27] and those with a cysteine-rich peptide tend to self-assemble into P particles [28], we evaluated the molecular size distributions of the three purified P domain proteins using a dynamic light scattering approach. The results (Figure 1C) showed that the P domain proteins from the GII.4 strain Cin3 self-assembled into P particles with a typical diameter of approximately 22 nm (Figure 1C, left panel, radius = 10.9 nm). The P particles formed by the GII.4 Sydney P domain protein appeared slightly smaller, with a diameter of 18.6 nm (Figure 1C, middle panel, radius = 9.3 nm). In contrast, the P domain protein from the GII.6 strain exhibited a predominant molecular diameter of approximately 5.9 nm (Figure 1C, right panel, radius = 2.9 nm), consistent with the molecular dimensions of the GII.4 P dimer (5.7 × 6.3 × 6.9 nm) as determined by crystallography approach (PDB code: 2OBS). The P dimer formation of this protein may be attributed to the removal of the arginine-cluster at the C-terminus of the P domain to enhance solubility (see Discussion). In all three cases, small amounts of aggregates were also observed.

### 3.2. Generation and Evaluation of Norovirus S Domain Proteins

The S domain protein from the GII.4 strain VA387 was expressed as a C-terminally Hisx6-tagged protein using a bacterial expression system and purified using Hisx6 tag-binding resin. SDS-PAGE analysis indicated that the purified protein had the expected molecular weight of approximately 25 kDa (Figure 1D). Gel-filtration chromatography (Figure 1E) showed that most of the S domain protein self-assembled into S particles [23], which were further confirmed by the electron microscopy (Figure 1F), consistent with the observation from our previous study [23].

### 3.3. Yolk IgY Production and Characterization

**Figure 2:**
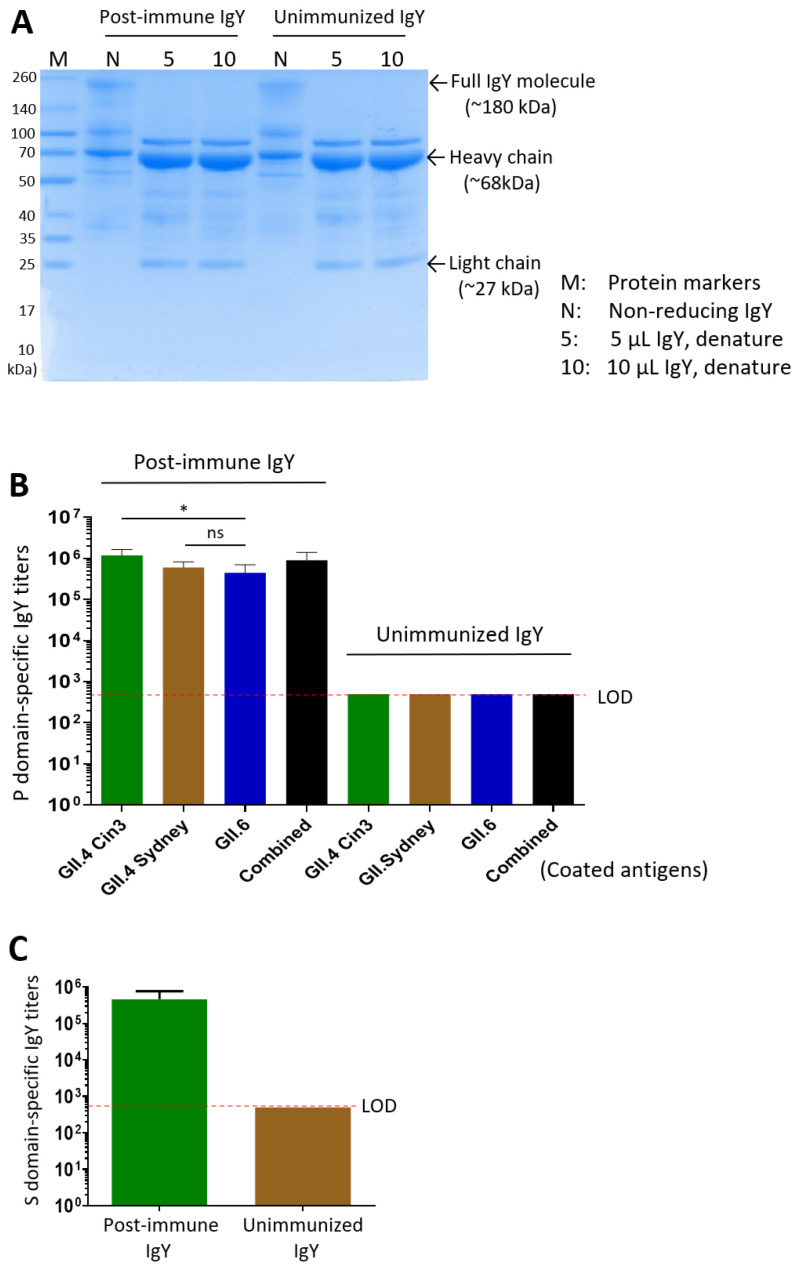
Characterization of yolk IgY following immunization with the P and S domain proteins. (**A**) SDS-PAGE analysis of IgY extracted from egg yolks after immunization with the P domain proteins (post-immune IgY) and from unimmunized control yolks (unimmunized IgY). Bands corresponding to the full IgY molecule (non-reducing) and its heavy and light chains (denatured) are indicated. (**B**) P domain-specific IgY antibody titers in the extracted IgY. The X-axis indicates the different capture antigens used for specific IgY titer determination. (**C**) S domain-specific IgY antibody titers in the post-immune IgY and unimmunized control IgY samples. Statistical differences between groups are indicated as non-significant (ns) when *p* > 0.05; significant (*) when *p* < 0.05, respectively.

Following immunization of laying hens with the three P domain proteins via three intramuscular doses, IgY antibodies were extracted from egg yolks. The extracted IgY yielded total protein concentrations of 1.76 mg/mL for the immunized preparation and 3.39 mg/mL for the unimmunized control. SDS-PAGE analysis of the IgY preparations revealed characteristic banding patterns. In the non-reduced samples, a distinct band at ~180 kDa was observed, corresponding to the full IgY molecule. In the denatured samples, a major band at ~68 kDa and a minor band at ~27 kDa were detected, representing the heavy and light chains, respectively (Figure 2A).

Using ELISA with the three purified P domain proteins as capture antigens, high titers of norovirus-specific antibodies were detected in the IgY preparations: 1:1,200,000 for the GII.4 Cin3 P domain, 1:600,000 for the GII.4 Sydney P domain, 1:450,000 for the GII.6 P domain, and 1:900,000 against the three combined antigens (Figure 2B). Similarly, following three intramuscular doses of the GII.4 norovirus S domain protein, the extracted yolk IgY, showing a total protein concentration of 2.38 mg/mL, exhibited an S domain-specific antibody titer of 1:460,000, while the unimmunized group showed no detectable S domain-specific IgY (Figure 2C).

### 3.4. Blockade of the IgY on Norovirus P Particle-HBGA Interaction

**Figure 3:**
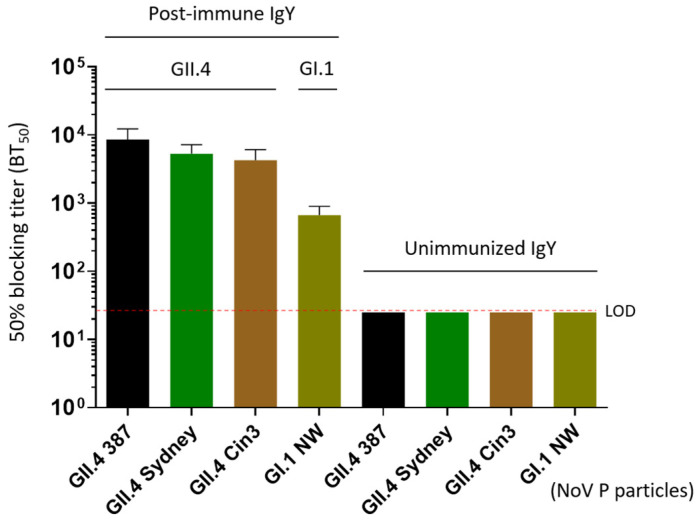
Blocking effects of the extracted IgY on various P particles representing homotypic (GII.4) and heterotypic (GI.1) noroviruses binding to their histo-blood antigen (HBGA) ligands in vitro. The X-axis indicated various P particles used in the blocking assays, whereas the Y-axis shows the 50% blocking titers (BT50) of the IgY against the binding of P particles and HBGA ligands. LOD refers to the limit of detection; NW refers to Norwalk virus.

Norovirus infection begins with the attachment of viral particles to HBGA glycans on the host cells. Human challenge studies and vaccine trials have demonstrated that pre-existing serum HBGA-blocking antibodies were associated with protection against norovirus infection and clinical disease [35,36,37]. The ability of antibodies to inhibit this interaction is considered a potential mechanism of neutralization. Using established blocking assays, we found that the extracted post-immune IgY preparations effectively inhibited the binding of P particles representing three homotypic GII.4 noroviruses to type A HBGA (Figure 3). Specifically, post-immune IgY exhibited 50% blocking titers (BT50) of 1:8533 to GII.4 VA387, 1:5333 to GII.4 Sydney, and 1:4266 to GII.4 Cin3. Notably, the post-immune IgY also demonstrated cross-reactive blocking activity, with a BT50 of 1:666 against the heterotypic GI.1 Norwalk virus. Due to the unavailability of a HBGA-binding GII.6 P particle or VLP, we were unable to determine the BT50 of the IgY against a homotypic GII.6 strain.

### 3.5. Neutralization Effects of the Post-Immune IgY on Norovirus Replication

**Figure 4:**
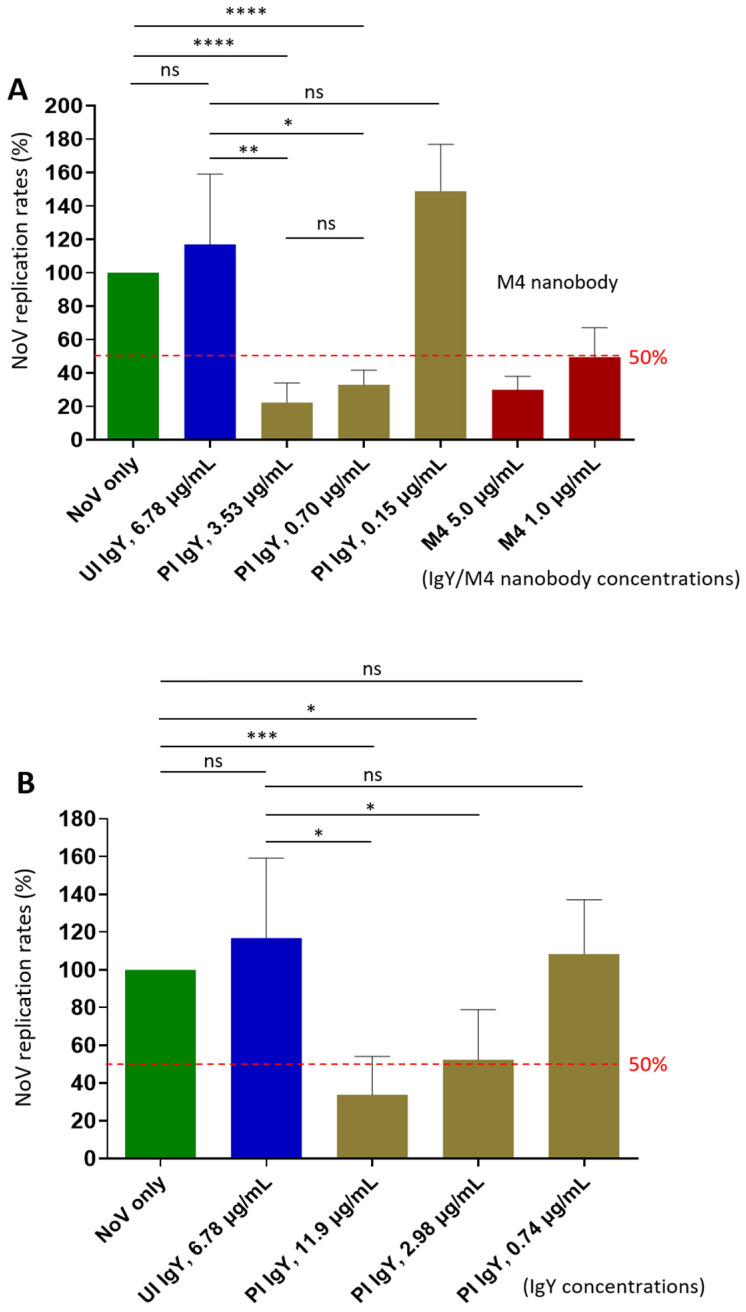
Neutralization activity of P domain- and the S domain-specific antibodies against replication of homotypic GII.4 Sydney norovirus in the human intestinal enteroid (HIE) system. (**A**) Neutralizing effects of extracted IgY raised against norovirus P domains on viral replication. The X-axis indicates the extracted IgY and control antibodies at indicated concentrations. The Y-axis shows virus replication rates of the treated viruses relative to the untreated virus control (no IgY, set as 100%). (**B**) Neutralizing activity of extracted IgY following immunization with the norovirus S domain protein against viral replication. The X-axis indicates the extracted IgY and control antibodies at various dilutions. The Y-axis shows norovirus replication rates of the treated viruses relative to the untreated virus control (no antibodies, set as 100%). In both experiments, the known M4 nanobody [34] at various concentrations was used as positive controls. Dashed lines indicate the 50% and 100% levels. UI IgY indicates unimmunized IgY; PI IgY indicates post-immune IgY. Statistical differences between groups are indicated as non-significant (ns) when *p* > 0.05; significant (*) when *p* < 0.05; highly significant (**) when *p* < 0.01; and extremely significant (*** or ****) when *p* < 0.001, or <0.0001, respectively.

Using the HIE system, the only available culture model that supports norovirus replication [25], we evaluated the neutralization effects of the IgY antibodies raised against the P or the S domains of the norovirus capsid protein. Our results showed that the IgY generated against the P domain inhibited replication of the homotypic GII.4 Sydney variant by 77.8% at a 1:500 dilution (3.53 µg/mL, *p* = 0.0049 vs. unimmune IgY) (Figure 4A). Even at 1:2500 dilution (0.70 µg/mL), the IgY still demonstrated 67.0% inhibition (*p* = 0.0208 vs. unimmune IgY). Based on these results, the 50% neutralization titer (NT50) of the IgY was estimated to exceed 1:2500. These findings support the potential application of IgY as a prophylactic and/or therapeutic agent against norovirus infection and disease. Considering that the P domain-specific IgY comprises approximately 3% to 10% of the total IgY, the polyclonal IgY antibodies appeared to exhibit higher neutralizing titer than that of the M4 nanobody (Figure 4A).

Notably, IgY raised against the S domain protein also inhibited norovirus replication in the HIE system (Figure 4B), showing inhibition rates of 66.2% and 48.5% at 1:200 (11.9 µg/mL, *p* = 0.0125) and 1:800 (2.98 µg/mL, *p* = 0.0423) dilutions, respectively. Based on these data, the NT50 of the S domain IgY was estimated to be approximately 1:800, which is lower than that (over 1:2500) of the IgY targeting the P domain. Nevertheless, these findings provide the first evidence that norovirus S domain contains neutralizing epitopes.

## 4. Discussion

Norovirus remains challenging to study, primarily due to the lack of a robust cell culture system for conventional virus propagation and the absence of an effective small animal model to simulate the disease process. These limitations, coupled with the genetic and antigenic diversity of human noroviruses, pose significant obstacles to vaccine development. Notably, recent Phase 2b clinical trials of the two most advanced norovirus vaccine candidates, the HIL-214 from HilleVax and VXA-GI.1-NN from Vaxart, demonstrated low protective efficacy [18,20]. These setbacks underscore the long road ahead toward an effective norovirus vaccine and highlight the need for alternative strategies to control and prevent norovirus infection and disease.

To address this, we explored in this study the use of yolk-derived IgY antibodies produced by immunizing laying hens with potential norovirus neutralizing antigens, specifically the P and S domains, which form the surface dimeric protrusions and the icosahedral inner shell of norovirus capsid, respectively. Our investigation provided evidence that IgY antibodies targeting either domain can effectively neutralize norovirus infection and replication in the HIE system. Notably, we present the first evidence that the norovirus S domain, which constitutes the viral shell, functions as a neutralizing antigen.

The P domain is recognized as a neutralizing antigen [38] because its ability to bind viral glycan receptors or attachment factors on the host cell surface and thereby initiate infection. However, the S domain has traditionally been considered a structural protein without a direct role in norovirus infection. While our findings demonstrate that the S domain acts as a neutralizing antigen, its mechanism of action remains unclear. In a previous study on feline calicivirus (FCV) [39], a virus closely related to norovirus within the Caliciviridae family, Conley and colleagues discovered that, following receptor engagement, the minor capsid protein VP2, associated with the VP1 S domains, forms a portal-like structure that facilitates the release of the viral RNA genome into the host cell cytosol [39]. This finding suggests that, beyond its structural role, the S domain contributes to the viral infection process. However, whether a similar scenario occurs in human norovirus remains to be determined.

Structurally, the norovirus shell is partially covered by multiple protrusions. In FCV, the portal structure assembles along the unique three-fold axis, with its pore located at the center of this axis in the capsid shell [39]. This region of the shell is also highly exposed, and therefore readily accessible to S domain-specific antibodies. We speculate that these antibodies may bind to and block this region, thereby inhibiting potential portal formation or a similar function, ultimately interfering with the viral infection process. Alternatively, the binding of S domain-specific antibodies to the exposed regions of the shell could induce conformational changes in the protrusions, potentially disrupting their functions. Further studies are needed to clarify the mechanism of action underlying the observed neutralizing activity of the S domain-targeting antibodies.

The identification of the S domain as a neutralizing antigen in this study raises an intriguing possibility for its application as a vaccine and antiviral target. Due to its highly conserved nature, the S domain may offer a promising solution to one of the major challenges in norovirus vaccine development, the extensive genetic and antigenic diversity among human noroviruses. Therefore, it would be valuable to investigate whether S domain antibodies can neutralize the replication of noroviruses representing different genotypes, and potentially even different genogroups, in the HIE system. Such data would provide critical evidence to support whether an inner shell-based norovirus vaccine is a viable direction for further pursuit.

We would like to point out a potential distinction between the S domain in current VLP-based norovirus vaccines and its potential use in an S particle-based vaccine. As observed with inactivated or hemagglutinin (HA)-based influenza vaccines, the host immune response is predominantly directed toward the immunodominant HA1 head, which, like the norovirus P domain, is highly variable among viral strains. In contrast, the immune response to the conserved HA2 stalk region of influenza virus, analogous to the norovirus S domain, is much weaker. This has led to ongoing efforts to develop a universal influenza vaccine that targets the conserved HA2 stem region while excluding the HA1 head [40,41,42]. We speculate that a similar scenario may apply to the VLP-based versus the S particle-based norovirus vaccines. This supports the idea that an S particle-based vaccine could offer broader and more effective protection against diverse norovirus strains, with a potentially lower risk of immune escape. Exploring this possibility will be the focus of our next phase of research.

As a low-cost alternative to traditional vaccine approaches, which are currently facing setbacks in recent Phase 2b clinical trials [18,20], IgY could be a promising strategy to prevent norovirus infection and potentially treat norovirus disease. Our finding that IgY antibodies targeting the two norovirus neutralizing antigens effectively inhibited viral infection and replication in the HIE system suggests that, when delivered to the gastrointestinal tract, where noroviruses infection occurs, either as formulated food supplements or oral capsules, these antibodies could bind and neutralize noroviruses, thereby preventing infection. Even after infection has occurred, the antibodies could bind to viral progeny, reducing reinfection within the intestine and decreasing active viral shedding in stool. This will ultimately reduce viral transmission and help prevent or limit the scale of outbreaks. Notably, this passive immunization approach could be especially valuable for immunocompromised patients, in whom vaccines may not elicit a sufficient immune response. Additionally, IgY antibodies could serve as a countermeasure for travelers who may require immediate protection, and in settings where norovirus outbreaks frequently occur, to treat the index cases and limit spread. Even when active immune-mediated protection by vaccines becomes available, a passive immunoprophylactic approach may be preferable for short-term travelers and those unable or unwilling to receive a vaccine.

The passive immunization approach may be especially applicable in norovirus infection, because the risk factors for acquiring the infection are largely predictable based on seasonality, demographics, and geographic location. Transient passive immunization during high-risk periods may be an appealing option for many people for whom the risk of infection is not constant, and for whom systemic immunization is either undesirable or unavailable.

During the production of norovirus P particles as immunogens for hen immunization, we removed the C-terminal arginine cluster (RRRAL) from the GII.6 P domain to increase the solubility of its P domain protein. However, this modification resulted in the inability of the P domain protein lacking the arginine-cluster to self-assemble into the 24-mer P particles (Figure 1C). Instead, it formed dimers and did not bind to HBGAs. Consequently, the IgY antibody titer specific to the GII.6 P domain was significantly lower than that specific to GII.4 Cin3 P domain (*p* = 0.0292), but not significantly different from that specific to GII.4 Sydney (*p* = 0.4136) (Figure 2B). In other words, the dimer form of the P domain protein appeared to be acceptable as an immunogen for generating neutralizing norovirus P domain-specific IgY antibodies.

A limitation of this study is our current inability to evaluate the neutralizing activity of our IgY antibodies against the homotypic GII.6 norovirus, as well as against heterotypic norovirus strains. We are in the process of obtaining live GII.6 virus and hope to conduct these experiments in the near future. Additionally, we were unable to generate GII.6 VLP and/or P particles that bind HBGAs, as a result, we could not determine blocking titer (BT50) of our IgY antibodies against this genotype. That said, our observed blocking titers against other homotypic and heterotypic noroviruses, along with the demonstrated neutralization activity against the homotypic GII.4 Sydney variant, support the overall concept and potential of our approach.

## 5. Conclusions

In this study we provide evidence to demonstrate that egg yolk-derived IgY antibodies targeting norovirus P or S domain effectively neutralize norovirus replication in the HIE system. These findings support the potential of the IgY antibodies as a scalable, cost-effective approach for preventing norovirus infection and disease.

## Data Availability

All relevant data has been published in this paper.

## Abbreviations

The following abbreviations are used in this manuscript:

IgY	Immunoglobulin Y
P domain	Protruding domain
S domain	Shell domain
VP1	Viral protein 1
HIE	Human intestinal enteroid
BT50	50% blocking titer
µg	Microgram
mL	Milliliter
AGE	Acute gastroenteritis
LMICs	Low- to middle-income countries
HICs	High-income countries
CDC	the Centers for Disease Control and Prevention
NoroSTAT	the Norovirus Sentinel Testing and Tracking
HBGAs	Histo-blood group antigens
SDS-PAGE	Sodium dodecyl sulfate polyacrylamide gel electrophoresis
BSA	Bovine serum albumin
DLS	Dynamic light scattering
FPLC	Fast Performance Liquid Chromatography
DTT	Dithiothreitol
KDa	Kilodalton
MDa	Megadalton
TEM	Transmission electron microscopy
PEG	Polyethylene glycol
BCA	Bicinchoninic acid
ELISA	Enzyme-linked immunosorbent assay
PBS	Phosphate-buffered saline
HRP	Horseradish peroxidase
OD	Optical density
GCDCA	Glycochenodeoxycholic acid
ORF	Open reading frame
NT50	50% neutralization titer
NIH	The National Institute of Health
IACUC	Institutional Animal Care and Use Committee
GST	Glutathione S-transferase
FCV	Feline calicivirus
VLP	Virus-like particle
HA	Hemagglutinin
